# Comparison of Rumen Fermentation Parameters and Microbiota of Yaks From Different Altitude Regions in Tibet, China

**DOI:** 10.3389/fmicb.2021.807512

**Published:** 2022-02-10

**Authors:** Lulu Han, Wanchao Xue, Hanwen Cao, Xiaoying Chen, Fasheng Qi, Tao Ma, Yan Tu, Qiyu Diao, Chengfu Zhang, Kai Cui

**Affiliations:** ^1^Key Laboratory of Feed Biotechnology of the Ministry of Agriculture and Rural Affairs, Institute of Feed Research of the Chinese Academy of Agricultural Sciences, Beijing, China; ^2^Animal Husbandry and Veterinary Station of Huangyuan, Huangyuan, China; ^3^State Key Laboratory of Hulless Barley and Yak Germplasm Resources and Genetic Improvement, Institute of Animal Science and Veterinary, Tibet Academy of Agricultural and Animal Husbandry Sciences, Lhasa, China; ^4^General Station of Animal Husbandry and Veterinary Technology Extension of Naqu, Naqu, China

**Keywords:** yak, altitude, region, rumen, microbiota

## Abstract

Rumen microbiota are closely linked to feed utilization and environmental adaptability of ruminants. At present, little is known about the influence of different extreme environments on the rumen microbiota of yaks. In this study, 30 ruminal fluid samples from 30 healthy female yaks (average 280 kg of BW) in 5–8 years of life were collected from three regions in Tibet, China, and compared by gas chromatography and high-throughput sequencing. Results showed that propionic acid, butyric acid, and total volatile fatty acids were significantly (*p* < 0.05) higher, while microbial abundance and diversity were significantly (*p* < 0.05) lower, in the Nagqu (4,500 m altitude) compared with the Xigatse (4,800 m altitude) and Lhasa (3,800 m altitude) regions. Principal coordinate analysis revealed significant (*p* < 0.05) differences in rumen microbial composition of yaks from different regions. Specifically, Bacteroidetes and Firmicutes were identified by linear discriminant analysis effect size (LDA > 3) as being the signature phyla for Xigatse and Nagqu regions, respectively. In addition, the relative abundance of *Rikenellaceae_RC9_gut_group*, *Quinella*, *Prevotellaceae_UCG-003*, *Lachnospiraceae_NK3A20_group*, *Papillibacter*, *Ruminococcaceae_UCG-010*, *Prevotellaceae_NK3B31_group*, and *Ruminococcaceae_UCG-005* correlated with altitude and rumen fermentation parameters (*p* < 0.05). Finally, the predicted function of rumen microbiota was found to differ between regions (*p* < 0.05). In summary, our results reveal that regions located at different altitudes influence microbiota composition and fermentation function of yaks’ rumen. The present findings can provide mechanistic insights on yak adaptation to high altitudes and improve the feeding efficiency of these animals in extreme regions.

## Introduction

Yaks (*Bos grunniens*) are long-haired ruminants living in high-altitude regions (>3,000 m) such as the Tibetan Plateau in China (Guo et al., 2015). About 90% of the world population of yaks is found in China (Li et al., 2018), where these iconic animals provide meat, milk, wool, and fuel for the local nomadic people (Liu et al., 2008; Long et al., 2008; Qiu et al., 2012; Gao et al., 2013; Zhang J. et al., 2020), who call yaks “boats of the plateau” (Liu et al., 2019).

Owing to the extreme environmental conditions of the Tibetan Plateau and long-term natural selection, yaks have developed a tolerance for cold, low oxygen, rough feeding, and hardship (Long et al., 2008; Qiu et al., 2012). Different environments lead to different metabolic strategies, which allow animals to adapt and minimize the adverse effects of environmental changes (Whiteley and Faulkner, 2005; Oliveira et al., 2021). Recent evidence suggests that such effect is largely due to the microbiota in the digestive tract, which play an important role in nutrient intake, metabolism, and immune response of the host (Furman et al., 2020; O’Hara et al., 2020; Bi et al., 2021). Consequently, the correlation between the adaptability of yaks to environmental changes and their gastrointestinal microbiota composition has attracted increasing attention. Guo et al. (2021) found that the fecal microbiota of yaks reflected seasonal dietary changes, allowing yaks to make better use of low-protein roughage. Whereas fecal microbiota composition of yaks differs across regions, its functional profile is more uniform (Liu et al., 2021). The rumen is one of the most important digestive organs of ruminants and is characterized by an abundance of microbiota (Guan et al., 2008; Khan et al., 2016). Indeed, owing to their microbiota, ruminants can ferment solid feed into volatile fatty acids (VFAs), which provide 70% of the required energy (Anantasook et al., 2013; Khan et al., 2016). At the same time, rumen microbiota and VFAs are the main factors explaining different feed efficiencies among individual cattle (Liang et al., 2017; Zhou et al., 2018; Zhang Y. et al., 2020). Although Wu et al. (2021) found that altitude affected the composition of rumen microbiota in yaks, it remains to be determined how microbiota and VFAs vary under extreme conditions (>3,000 m altitude).

To understand the response of yak rumen microbiota and VFAs to different altitudes, the present study employed microbiome technology to characterize rumen composition in yaks living at different altitudes in three extreme regions of Tibet. We hypothesized that rumen fermentation parameters and microbiota of yaks would change dynamically with increasing altitude.

## Materials and Methods

The experimental protocol was approved by the Chinese Academy of Agricultural Sciences Animal Ethics Committee, and all procedures were performed in accordance with humane animal care and handling (AEC-CAAS-20190905).

### Study Regions, Animals, and Experimental Design

Three study regions, including Zhongba County, Xigatse City (84°03′ E, 29°77′ N; about 4,800 m altitude, HAL), Nagqu City (92°07′ E, 31°48′ N; about 4,500 m altitude, MAL), and Dangxiong County, Lhasa City (91°05′ E, 30°51′ N; about 3,800 m altitude, LAL), were selected. The three regions are key breeding grounds for yaks; their average annual air temperature is −4.0°C, −0.5°C, and 2.0°C, while precipitation is 280, 433, and 485 mm, respectively.

In September 2019, ten healthy female yaks (average 280 kg of BW) in 5–8 years of life were randomly selected from every study region. At the study regions, yaks are allowed to live free-range, follow their dams, and are not given any artificial feed, relying instead on typical alpine meadows as the main type of vegetation.

### Ruminal Chyme Collection and Analysis of Fermentation Parameters

Before morning grazing, ruminal chyme (30 samples in total) was collected through the mouth of yaks using an esophageal tube as described previously (Ramos-Morales et al., 2014). Next, the chyme was filtered through a four-layer woven gauze to collect the ruminal fluid, which was immediately frozen in liquid nitrogen (−80°C) for subsequent microbiota and fermentation characterization. The pH value was detected using a portable pH meter (206-pH1; Testo). For VFA analysis, three tubes containing 1 ml of rumen fluid were taken from each sample after thawing and centrifugation at 20,000 × *g* and 4°C for 15 min. Then, 0.25 ml metaphosphoric acid (25 g/100 ml) was added per tube and the sample was analyzed by gas chromatography equipping a megabore HP-MOLSIV column (film thickness: 30 m × 0.53 mm × 25 μm) (SP-3420A; Beifenrili Analyzer Associates, Beijing, China) (Yue et al., 2009).

### DNA Extraction, PCR Amplification, and 16S rRNA Sequencing

Microbial DNA was extracted from rumen fluid samples (30 samples in total) using PowerSoil DNA Isolation Kit (MoBio Laboratories, Carlsbad, CA) following the manufacturer’s guidelines. The concentration and purity of the extracted DNA were measured by a NanoDrop 2000 Spectrophotometer (Thermo Fisher Scientific, Waltham, MA, United States). The V3–V4 region of the bacterial 16S rRNA gene was amplified using primer 338F (5′-ACTCCTACGGGAGGCAGCAG-3′) and 806R (5′-GGACTACHVGGGTWTCTAAT-3′) (Takahashi et al., 2014). PCR amplification proceeded through an initial denaturing step at 94°C for 5 min; 28 cycles at 94°C for 30 s, 55°C for 30 s, and 72°C for 60 s; and a final extension at 72°C for 7 min using 25-μl reaction volumes, containing 12.5 μl 2 × Taq PCR MasterMix, 3 μl BSA (2 ng/μl), 1 μl forward primer (5 μM), 1 μl reverse primer (5 μM), 2 μl template DNA, and 5.5 μl ddH2O. The PCR products were detected by 2% agarose gel electrophoresis, purified with the Agencourt AMPure XP kit (Beckman Coulter, La Brea, CA, United States) according to the manufacturer’s instructions, and quantified by QuantiFluor-ST (Promega, Madison, WI, United States). Purified amplicons were sequenced on an Illumina MiSeq-PE300 platform (Illumina Inc., San Diego, CA, United States), generating 2 × 300 bp paired-end reads.

### Sequence Analysis

The obtained paired-end reads in the original DNA fragments were merged using Flash version 1.20 (Mago and Salzberg, 2011), and then each sample was separated according to a unique barcode. After removing barcodes, primers, and splice variants, raw reads were obtained. To generate high-quality reads, a specific sliding window strategy was adopted for Trimmomatic version 0.36, the window size was set to 50 bp, the average quality value was 20, and the minimum reserved sequence length was 120 bp; in addition, sequences containing N were removed by Pear version 0.9.6 (Bolger et al., 2014). Afterward, Flash and Pear were used to merge the sequences at both ends according to PE overlap correlation, the minimum overlap was set to 10 bp, and the mismatch rate was 0.1, allowing for fasta sequences to be obtained. Finally, the chimera containing fasta sequences was eliminated by comparison with the Gold database using the UCHIME algorithm, whereas the unknown database was removed by *de novo* means (Knight, 2011). Simultaneously, the short sequences, failing to meet the requirements, were removed, while high-quality sequences of clean reads were obtained. Subsequently, these clean tags were clustered into operational taxonomic units (OTUs) based on a 97% sequence similarity threshold using the UPARSE algorithm in Vsearch version 2.7.1. In each OTU, the richest sequence was filtered as the representative sequence (Edgar, 2013). To derive species classification information corresponding to each OTU, the representative sequences were compared and analyzed by the RDP Classifier algorithm version 2.2 (Wang, 2007) and Silva database^1^ (Quast et al., 2012), allowing community annotation at kingdom, phylum, class, order, family, and genus level. After the sample number of the lowest sequence was flattened, alpha diversity (Chao1, Shannon, Simpson, and observed species) was calculated by QIIME 2, while intergroup alpha index variability was demonstrated by the Kruskal–Wallis test in R version 4.0.2 (Miller et al., 2016). Principal coordinate analysis (PCoA) based on Bray–Curtis dissimilarity matrices and QIIME 2 was used to calculate differences in bacterial communities among groups. The linear discriminant analysis effect size (LEfSe, LDA > 3) was used to identify significant bacteria among the three groups (Miller et al., 2016). PICRUSt2 software was used to predict microbiota function and explore differences among the three groups (Douglas et al., 2019).

Rumen fermentation parameters were analyzed using one-way ANOVA and Duncan’s multiple comparison in SPSS 22.0 software (IBM, Chicago, IL, United States). Differences in alpha diversity, relative abundance at phylum, family, and genus level, as well as microbiota function among the three groups were tested using the Kruskal–Wallis method in R version 4.0.3. PCoA, Venn, column, and LEfSe results were visualized using the “ape” and “ggplot2,” “limma,” “ggplot2,” and “ggtree” packages in R, respectively. The “corrplot” package in R was used to analyze the correlation between the top 20 genera of all samples, rumen fermentation parameters, and altitude (based on Spearman’s coefficient). The network containing the top 20 genera was visualized using the “igraph” package in R version 4.0.3. All data are reported as means, and intergroup differences of *p* < 0.05 were considered significant.

## Results

### Rumen Fermentation Parameters of Yaks Living in Different Regions

As reported in Table 1, rumen fermentation parameters differed significantly between the three groups (*p* < 0.05), whereas the pH value had no difference between the three groups (*p* > 0.05). Also, the MAL group exhibited the highest (*p* < 0.05) total VFA content and molar proportions of propionic and butyric acids, whereas the molar proportion of acetic acid and A/P (acetic acid/propionic acid) were lower (*p* < 0.05) than in other groups. Interestingly, rumen fermentation parameters were similar between HAL and LAL groups (*p* > 0.05).

**TABLE 1:** Effect of regions located at distinct altitudes on yak rumen fermentation parameters.

Parameters	Groups^1^			SEM	*P*-value
	HAL	MAL	LAL		
PH	6.41	6.54	6.45	0.042	0.834
T-VFA (mmol/L)	49.62^*b*^	69.41^*a*^	45.22^*b*^	2.943	< 0.001
Acetic acid (%)	74.58^*a*^	61.73^*b*^	72.09^*a*^	0.011	< 0.001
Propionic acid (%)	15.88^*b*^	24.46^*a*^	18.21^*b*^	0.008	< 0.001
Butyric acid (%)	9.54^*b*^	13.81^*a*^	9.70^*b*^	0.005	< 0.001
A/P	4.73^*a*^	2.57^*b*^	3.97^*a*^	0.18	< 0.001

*^1^HAL, high-altitude region (Zhongba County, Xigatse City; 4,800 m); MAL, medium-altitude region (Nagqu City; 4,500 m); LAL, low-altitude region (Dangxiong County, Lhasa City; 3,800 m). T-VFA, total volatile fatty acids; A/P, acetic acid/propionic acid.*

*Different superscript letters in the same row denote significant differences (p < 0.05).*

### Taxonomic Composition of Rumen Bacteria

**FIGURE 1:**
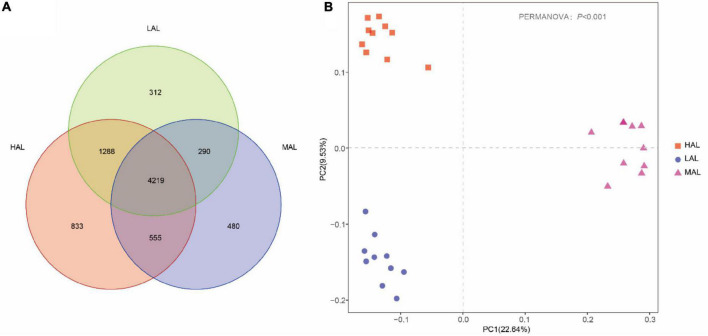
Response of rumen microbiota from yaks among the three regions. **(A)** Composition of rumen microbiota from yaks inhabiting regions located at distinct altitudes (OTU-level analysis). **(B)** PCoA analysis of rumen microbiota from yaks inhabiting regions located at distinct altitudes. HAL, high-altitude region (Zhongba County, Xigatse City; 4,800 m); MAL, medium-altitude region (Nagqu City; 4,500 m); LAL, low-altitude region (Dangxiong County, Lhasa City; 3,800 m).

Gradual stabilization of the OTU rank curve chart indicates high coverage of test samples (Supplementary Figure 1). The rumen microbiota consisted of 5,491,884 high-quality reads, with an average of 183,063 reads per sample. A total of 7,977 OTUs were obtained based on 97% nucleotide sequence identification: 6,895 OTUs in HAL, 5,544 in MAL, and 6,109 in LAL groups (Figure 1A). Of these, 4,219 OTUs (52.89% of the total) were shared among samples from different groups. Alpha diversity calculations (Table 2) revealed significantly higher indices of Shannon and community richness (Chao1 and observed species) in HAL and LAL groups compared with the MAL group (*p* < 0.05), whereas no significant difference was detected between HAL and LAL groups (*p* > 0.05). Interestingly, we found Simpson index in HAL significantly higher (*p* < 0.05) than MAL group, whereas no significant difference was detected between LAL and other groups (*p* > 0.05). PCoA plots of bacterial structure profiles (Figure 1B) revealed distinct clustering of the three regions. The segregation and dissimilarities observed at OTU level were revealed using Bray–Curtis matrices (PERMANOVA, *p* < 0.001).

**TABLE 2:** Effect of regions located at distinct altitudes on alpha diversity of yak rumen microbiota.

Indices	Groups^1^			SEM	*P*-value
	HAL	MAL	LAL		
Chao1	4373*^a^*	3431.5*^b^*	4203.7*^a^*	168.51	<0.001
Observed_species	3408.6*^a^*	2621.1*^b^*	3234.6*^a^*	157.28	<0.001
Shannon	9.28*^a^*	8.53*^b^*	9.19*^a^*	0.27	<0.001
Simpson	0.994*^a^*	0.987*^b^*	0.991*^ab^*	0.003	<0.001

*^1^HAL, high-altitude region (Zhongba County, Xigatse City; 4,800 m); MAL, medium-altitude region (Nagqu City; 4,500 m); LAL, low-altitude region (Dangxiong County, Lhasa City; 3,800 m).*

*Different superscript letters in the same row denote significant differences (p < 0.05).*

**FIGURE 2:**
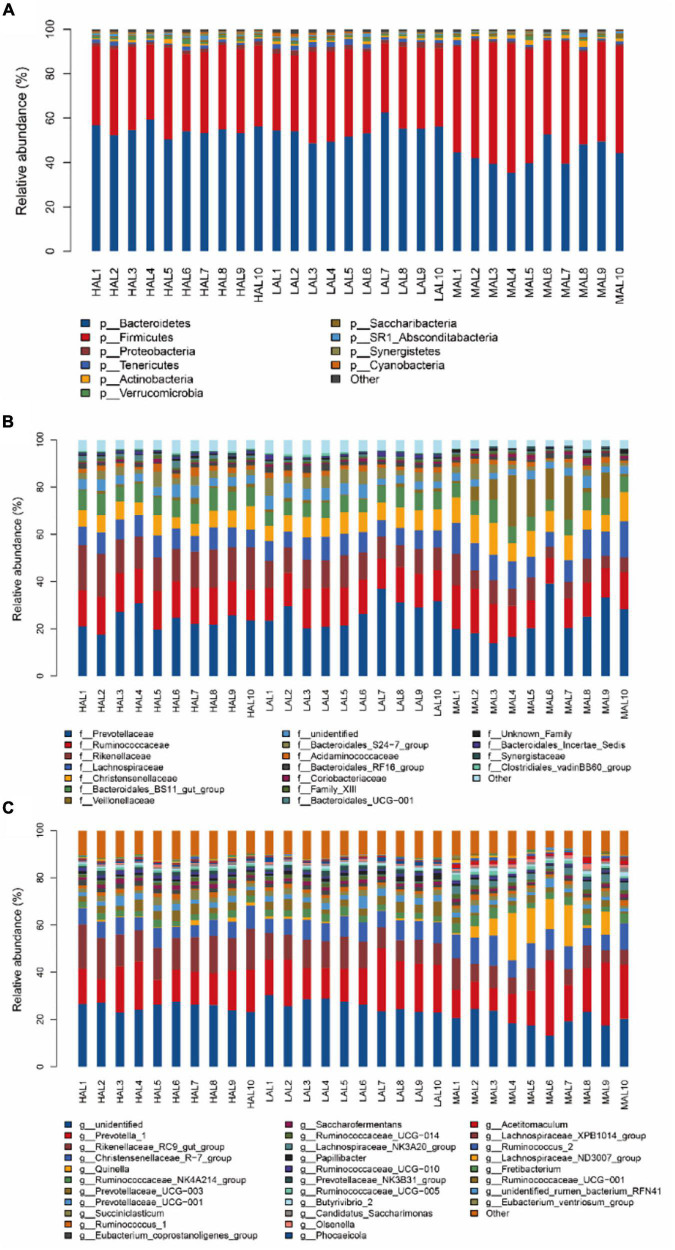
Compositional profiles of rumen microbiota from yaks inhabiting regions located at distinct altitudes. **(A)** Phylum level, **(B)** family level, and **(C)** genus level. HAL, high-altitude region (Zhongba County, Xigatse City; 4,800 m); MAL, medium-altitude region (Nagqu City; 4,500 m); LAL, low-altitude region (Dangxiong County, Lhasa City; 3,800 m).

Next, we proceeded with bacterial identification of the 30 samples at different levels. At the phylum level, the top five ruminal bacteria of yaks across all samples were Bacteroidetes (50.69%), Firmicutes (41.11%), Proteobacteria (1.45%), Tenericutes (1.40%), and Actinobacteria (1.05%) (Figure 2A and Supplementary Table 1). The MAL group presented the lowest (*p* < 0.05) relative abundances of Bacteroidetes and Verrucomicrobia, but the highest (*p* < 0.05) relative abundance of Firmicutes compared with the HAL and LAL groups. At the family level, Prevotellaceae (24.67%) was the most abundant taxon, followed by Ruminococcaceae (14.76%) and Rikenellaceae (11.67%) across all samples; other families included Lachnospiraceae (9.15%), Christensenellaceae (8.42%), Bacteroidales_BS11_gut_group (7.20%), Veillonellaceae (4.05%), and Bacteroidales_S24-7_group (3.44%) (Figure 2B and Supplementary Table 2). Notably, the relative abundances of Lachnospiraceae, Christensenellaceae, and Veillonellaceae were higher (*p* < 0.05) in the MAL compared with other groups, whereas the relative abundance of Rikenellaceae increased significantly (*p* < 0.05) according to the following order: MAL < LAL < HAL. At the genus level, the dominant genera across all groups were *Prevotella_1* (16.82%), *Rikenellaceae_RC9_gut_group* (11.26%), and *Christensenellaceae_R-7_group* (8.06%) (Figure 2C and Supplementary Table 3). Specifically, the MAL group displayed the highest (*p* < 0.05) relative abundances of *Christensenellaceae_R-7_group*, *Ruminococcaceae_NK4A214 _group*, *Quinella*, *Ruminococcaceae_UCG-005*, *Olsenella*, *Butyrivibrio_2*, *Lachnospiraceae_NK3A20_group*, and *Acetitomaculum*, but the lowest (*p* < 0.05) relative abundances of *Prevotellaceae_UCG-003*, *Rikenellaceae_RC9_gut_group*, *Eubacterium_coprostanoligenes_group*, *Saccharofermentans*, and *Prevotellaceae_NK3B31_group* compared with the LAL and HAL groups. The relative abundance of *Ruminococcaceae_UCG-010* was significantly (*p* < 0.05) higher in the HAL group compared with the MAL and LAL groups, whereas the relative abundance of *Papillibacter* was higher (*p* < 0.05) in the LAL group than in the other groups.

**FIGURE 3:**
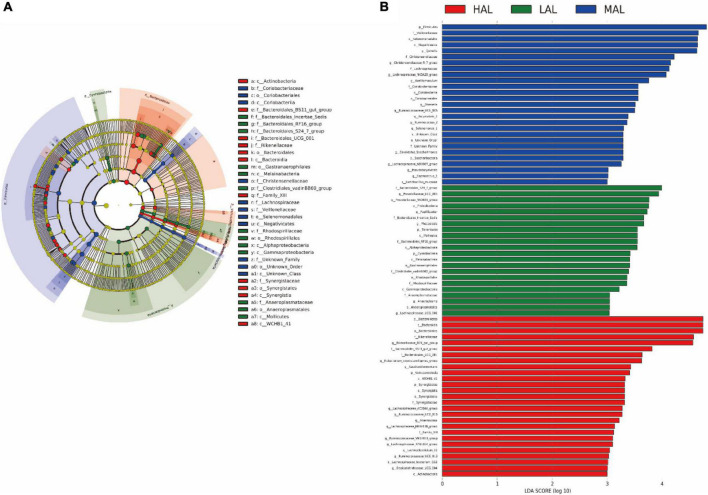
LEfSe analysis of rumen microbiota from yaks inhabiting regions located at distinct altitudes. **(A)** Histogram of linear discriminant analysis scores based on Classification Information. **(B)** Linear discriminant analysis effect size cladogram based on Classification Information. HAL, high-altitude region (Zhongba County, Xigatse City; 4,800 m); MAL, medium-altitude region (Nagqu City; 4,500 m); LAL, low-altitude region (Dangxiong County, Lhasa City; 3,800 m).

To better understand the dominance of specific bacteria within the three groups, we used the LEfSe method (Figure 3). The Firmicutes and Saccharibacteria phyla, including genera *Quinella*, *Christensenellaceae_R_7_group*, *Lachnospiraceae _NK3A20_group*, *Acetitomaculum*, *Olsenella*, *Ruminococcaceae _UCG_005*, *Butyrivibrio_2*, and *Ruminococcus_2*, were abundant in the MAL group. The Bacteroidetes, Verrucomicrobia, and Synergistetes phyla, including *Rikenellaceae_RC9_gut _group*, *Saccharofermentans*, *Lachnospiraceae_AC2044_group*, *Ruminococcaceae_UCG_010*, *Anaerovorax*, and *Family_XIII*, were enriched in the HAL group. Finally, the Papillibacter, Tenericutes, Cyanobacteria, and Bacteroidales_S24_7_group phyla, including *Prevotellaceae_UCG_003*, *Prevotellaceae_NK3 B31_group*, *Papillibacter*, *Anaeroplasma*, and *Lachnospiraceae _UCG_006*, were over-represented in the LAL group.

### Rumen Fermentation Parameters and Altitude Correlate With Bacterial Communities

**FIGURE 4:**
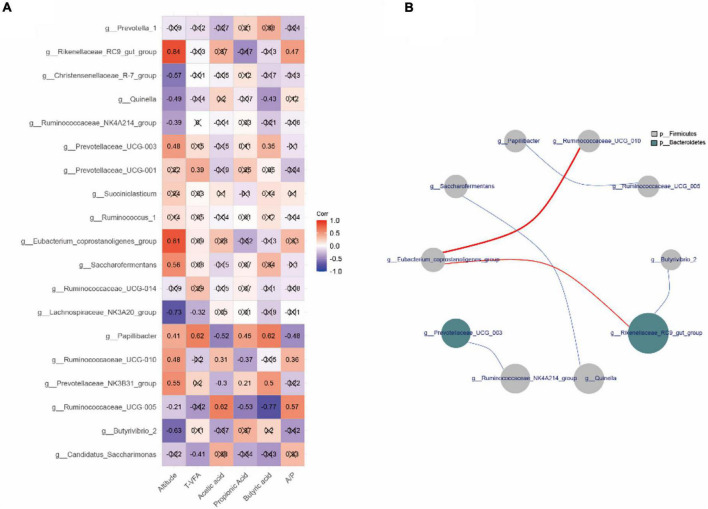
Correlation analysis of rumen genera in yaks inhabiting regions located at distinct altitudes. **(A)** Correlation between the top 20 relative abundances of genera, fermentation parameters, and altitude. **(B)** Interaction among the top 20 relative abundances at genus level. Red represents a positive correlation and blue a negative correlation. The number represents the correlation coefficient. The × sign represents *p* > 0.05. The size and color of the circle represent the relative abundance of the respective genus and the phylum.

The correlation between relative abundance of the top 20 bacterial genera, fermentation parameters, and altitude (Figure 4A) revealed that the relative abundances of *Rikenellaceae_RC9_gut_group*, *Quinella*, *Prevotellaceae_UCG-003*, *Lachnospiraceae_NK3A20_group*, *Papillibacter*, *Ruminococcaceae_UCG-010*, *Prevotellaceae_NK3B31_group*, and *Ruminococcaceae_UCG-005* correlated with altitude and rumen fermentation (*p* < 0.05). For example, the relative abundance of *Rikenellaceae_RC9_gut_group* correlated positively with altitude (*r* = 0.84) and A/P (*r* = 0.47), whereas the relative abundance of *Quinella* correlated negatively with altitude (*r* = −0.49) and butyric acid (*r* = −0.43). The relative abundance of *Papillibacter* correlated positively with altitude (*r* = 0.41), total VFA (*r* = 0.62), propionic acid (*r* = 0.45), and butyric acid (*r* = 0.62), but negatively with acetic acid (*r* = −0.52) and A/P (*r* = −0.48). Interestingly, we found a significant negative correlation between *Papillibacter* and *Ruminococcaceae_UCG-005* (|*r*|> 0.6, *p* < 0.05; Figure 4B).

### PICRUSt2 Function Prediction

The function of rumen microbial communities in yaks from three different regions was predicted by PICRUSt2 software and related to KEGG pathways (Table 3). Notably, “Biosynthesis of ansamycins” (4.47%) was the most abundant pathway in all three groups, followed by “Biosynthesis of vancomycin group antibiotics” (2.47%), “Valine, leucine and isoleucine biosynthesis” (2.25%), and “D-Glutamine and D-glutamate metabolism” (2.19%). The Kruskal–Wallis test confirmed that a total of 25 pathways showed significant (*p* < 0.05) differences among the three groups (Table 3). Specifically, “Amino acid metabolism,” “D-Alanine metabolism,” and “Thiamine metabolism” pathways, as well as “Carbohydrate metabolism,” “Cell motility,” and “Folding, sorting and degradation” gene categories were significantly (*p* < 0.05) more abundant in the MAL than in the other two groups.

**TABLE 3:** Functional predictions for rumen microbiota in yaks inhabiting regions located at distinct altitudes; only significantly different KEGG pathways are shown.

KEGG_Pathways			Groups^1^			SEM	*P*-value
Level I	Level II	Level III	HAL	MAL	LAL		
**Metabolism**							
**Metabolism of terpenoids and polyketides**							
Biosynthesis of ansamycins			4.51*^a^*	4.63*^a^*	4.26*^b^*	0.052	0.0113
Biosynthesis of vancomycin group antibiotics			2.53*^a^*	2.37*^b^*	2.50*^a^*	0.015	<0.001
Terpenoid backbone biosynthesis			1.40*^b^*	1.42*^a^*	1.40*^b^*	0.002	0.0112
**Amino acid metabolism**							
Valine, leucine, and isoleucine biosynthesis			2.23*^b^*	2.29*^a^*	2.22*^b^*	0.007	<0.001
Lysine biosynthesis			1.52*^b^*	1.59*^a^*	1.53*^b^*	0.003	<0.001
Phenylalanine, tyrosine, and tryptophan biosynthesis			1.28*^b^*	1.33*^a^*	1.29*^b^*	0.004	<0.001
Histidine metabolism			1.26*^a^*	1.26*^a^*	1.25*^b^*	0.002	0.0012
Cysteine and methionine metabolism			1.22*^b^*	1.28*^a^*	1.23*^b^*	0.005	<0.001
**Metabolism of other amino acids**							
D-Glutamine and D-glutamate metabolism			2.17*^b^*	2.21*^a^*	2.18*^ab^*	0.006	0.0393
D-Alanine metabolism			1.60*^b^*	1.65*^a^*	1.60*^b^*	0.006	<0.001
Selenocompound metabolism			1.06*^a^*	1.02*^c^*	1.03*^b^*	0.004	<0.001
**Metabolism of cofactors and vitamins**							
Pantothenate and CoA biosynthesis			1.86*^b^*	1.90*^a^*	1.86*^b^*	0.006	0.0125
Thiamine metabolism			1.50*^b^*	1.60*^a^*	1.53*^b^*	0.006	0.0016
Folate biosynthesis			1.25*^a^*	1.15*^b^*	1.24*^a^*	0.011	<0.001
Nicotinate and nicotinamide metabolism			1.12*^b^*	1.11*^b^*	1.14*^a^*	0.005	0.0178
Lipoic acid metabolism			1.12*^b^*	1.03*^b^*	1.18*^a^*	0.020	0.0195
**Carbohydrate metabolism**							
C5-Branched dibasic acid metabolism			1.69*^b^*	1.80*^a^*	1.68*^b^*	0.015	0.0063
Pentose phosphate pathway			1.52*^b^*	1.57*^a^*	1.51*^b^*	0.010	0.0015
Citrate cycle (TCA cycle)			1.17*^a^*	1.10*^c^*	1.15*^b^*	0.006	<0.0001
Amino sugar and nucleotide sugar metabolism			1.03*^b^*	1.07*^a^*	1.04*^b^*	0.004	0.0145
**Lipid metabolism**							
Fatty acid biosynthesis			1.74*^a^*	1.65*^b^*	1.69*^b^*	0.011	0.0153
**Energy metabolism**							
Carbon fixation pathways in prokaryotes			1.29*^a^*	1.25*^b^*	1.27*^b^*	0.004	<0.001
**Cellular processes**							
**Cell motility**							
Bacterial chemotaxis			1.112*^b^*	1.505*^a^*	1.130*^b^*	0.039	<0.001
**Genetic information processing**							
**Folding, sorting, and degradation**							
Protein export			1.486*^b^*	1.503*^a^*	1.485*^b^*	0.007	0.0032
Replication and repair							
DNA replication			1.279*^a^*	1.263*^b^*	1.272*^ab^*	0.002	0.0249

*^1^HAL, high-altitude region (Zhongba County, Xigatse City; 4,800 m); MAL, medium-altitude region (Nagqu City; 4,500 m); LAL, low-altitude region (Dangxiong County, Lhasa City; 3,800 m).*

*Different superscript letters in the same row denote significant differences (p < 0.05).*

## Discussion

This is the first study to evaluate the influence of extreme environments, corresponding to different altitudes of the Tibetan Plateau, on rumen microbiota in yaks. The study highlights how rumen fermentation, bacterial composition, and function are related to the regions in which the yaks live.

Ruminants rely on VFAs produced in the rumen during fermentation as a source of energy. Indeed, their feed efficiency is related to such VFAs (Lam et al., 2018; Zhou et al., 2018). While propionic acid can supply additional energy to ruminants, acetic and butyric acids are converted to methane and carbon dioxide during the consumption process, lowering energy efficiency (Gunun et al., 2018). Propionic acid and butyric acid compete with methane for hydrogen during fermentation, reducing methane energy consumption while improving energy supply (Pragna et al., 2018). Acetic acid and butyric acid originate mainly from the fermentation of fiber, whereas propionic acid derives from the fermentation of sugar and starch (Astawa et al., 2011). In this study, VFAs and propionic acid were highest in the rumen of MAL animals, suggesting that feed efficiency might be better in this than in the other groups. Contrary to the present results, Zhang et al. (2016) found that ruminants living at elevated altitude had higher VFA content and significantly upregulated genes responsible for VFA absorption in the rumen. Diet is the most important factor regulating development and colonization of rumen microbiota (Zhou et al., 2018; Lin et al., 2019; Myer, 2019). Ram (2005) reported that meadows found at different elevation in the Uttaranchal Himalaya exhibited distinct species richness. Similarly, Bao et al. (2021) linked the alpine meadows between Dangxiong and Nagqu regions of Tibet with a varied nutritional value. Regional variation could be related to temperature differences between day and night, photosynthesis, and topography (Han et al., 1997; Skapetas et al., 2004). Accordingly, further experiments are required to ascertain whether the observed differences are due to the extreme climatic conditions and altitude tested in this study or to the quality of grass in the different regions. Furthermore, the alpha and beta diversity indices of rumen microbiota differed significantly between the three groups, indicating a close relationship between the diversity of rumen microbiota in yaks and geographic location. Interestingly, unlike VFAs, the diversity of ruminal microbiota first decreased and then increased with elevation. Shabat et al. (2016) found that microbiota diversity correlated negatively with feed efficiency, with efficient dairy cattle exhibiting lower rumen microbiota diversity, methane emission, and acetic acid concentration, but higher propionic acid content. Taking into account those and the present results, it appears that yaks in the MAL group are more efficient at utilizing their pasture and emit less methane.

As observed previously (Zhang et al., 2016; Guo et al., 2021; Liu et al., 2021), Bacteroidetes and Firmicutes were the dominant taxa identified in this study. Guo et al. (2020) reported that Bacteroidetes were the most important bacteria in yak rumen from birth to adulthood. Here, we found that Firmicutes were the signature bacteria of the MAL group as they were significantly more abundant than in the other two groups. Because Firmicutes play an important role in energy conversion (Turnbaugh et al., 2006), our results indicated that they might be the key enablers of high feed utilization by yaks in the MAL group. Furthermore, the relative abundance of *Papillibacter* was found to correlate positively with altitude, whereas *Ruminococcaceae_UCG_005* showed a negative correlation. Mao et al. (2013) found that dairy cattle had lower levels of *Papillibacter* when in a state of subacute ruminal acidosis, which indirectly suggests that yaks may be better suited to living at higher altitudes. *Ruminococcaceae_UCG_005* enterotype has been associated with yak diets rich in proteins but low in fibers (Guo et al., 2021). Combined with the correlation results of VFAs, we hypothesize that *Papillibacter and Ruminococcaceae_UCG_005* may be the hub genera allowing for adaptation to high altitude. By antagonizing each other, they may allow yaks to adjust to different altitudes through modulation of the ruminal VFA content. The genera *Rikenellaceae_RC9_gut_group*, *Quinella*, *Prevotellaceae_UCG-003*, *Lachnospiraceae_NK3A20_group*, *Prevotellaceae_NK3B31_group*, and *Ruminococcaceae_UCG-005* exhibited a correlation with different altitudes and rumen fermentation parameters, suggesting that they, too, may play a key part in the adaptation to extreme environments. As there are other reports pointing to the involvement of altitude-related genera in VFA production, rumen development, immunity, and methane output (Petri et al., 2013; Zhai et al., 2017; Pragna et al., 2018; Lin et al., 2019; Sha et al., 2021), we believe that altitude affects the microbial composition of yaks’ rumen and enables adaptation to extreme environments by modulating rumen fermentation products.

The functional properties of microbiota dictate the host–microbiome interaction (Lin et al., 2019; Myer, 2019). Here, the PICRUSt2 result revealed that the function of yak rumen microbiota differed between the three regions. In contrast, Liu et al. (2021) found a similar functional gene composition of yak intestinal microbes across the three regions, suggesting that the yak rumen may be more sensitive than the intestinal tract in terms of environmental adaptability. Due to limited experimental conditions, this study did not further explore how diet and extreme environment affected microbiota composition beyond the correlation with altitude. The mechanism regulating adaptation between yak genome and extreme environment has been described previously (Qiu et al., 2012), and the microbiome is closely related to animal activities (Wu et al., 2011). Therefore, future metagenomics studies may help untangle the contribution of gut microorganisms to mammalian adaptation to extreme environments.

## Conclusion

This study has analyzed the rumen microbiota and fermentation products of yaks living at different altitudes in Tibet, China. Indeed, rumen fermentation, composition, and function were found to vary across regions, with some genera correlating strongly with altitude. Based on the results, we found the middle-altitude region was suitable for rumen fermentation end-products and microbiota composition compared with the low- or high-altitude regions in Tibet. These results provide an insight on the mechanism enabling yaks to adapt to high altitude and maximize feed efficiency in extreme environments.

## Data Availability Statement

The datasets presented in this study can be found in online repositories. The names of the repository/repositories and accession number(s) can be found below: https://submit.ncbi.nlm.nih.gov/subs/, PRJNA776716.

## Ethics Statement

The animal study was reviewed and approved by the Chinese Academy of Agricultural Sciences Animal Ethics Committee approved the experimental protocol, and all the methods conducted in this experiment were in accordance with humane animal care and handling procedures (AEC-CAAS-20190905). Written informed consent was obtained from the owners for the participation of their animals in this study.

## Author Contributions

WX: conceptualization. KC: methodology, supervision, and funding acquisition. LH: software, resources, data curation, and writing—original draft preparation. YT, QD, and XC: validation. HC: formal analysis and visualization. FQ: investigation. LH, WX, TM, KC, and CZ: writing—review and editing. KC and CZ: project administration. All authors contributed to the article and approved the submitted version.

## Conflict of Interest

The authors declare that the research was conducted in the absence of any commercial or financial relationships that could be construed as a potential conflict of interest.

## Publisher’s Note

All claims expressed in this article are solely those of the authors and do not necessarily represent those of their affiliated organizations, or those of the publisher, the editors and the reviewers. Any product that may be evaluated in this article, or claim that may be made by its manufacturer, is not guaranteed or endorsed by the publisher.

## Footnotes

1
http://www.arb-silva.de
