# Supplementary material for: Comparison of Rumen Fermentation Parameters and Microbiota of Yaks From Different Altitude Regions in Tibet, China
Source: Front Microbiol. 2022 Feb 10;12:807512. doi: 10.3389/fmicb.2021.807512 (PMC8867021; doi:10.3389/fmicb.2021.807512)
Supplement: Supplementary file 1 [file Data_Sheet_1.docx]

**Supplementary Table 1.** Composition of rumen microbiota at the phylum level.

| Phylum | Groups^1^ | | | SEM | *P*-value |
| --- | --- | --- | --- | --- | --- |
|  | HAL | MAL | LAL |  |  |
| Bacteroidetes | 54.528^a^ | 43.531^b^ | 54.031^a^ | 2.3708 | <0.001 |
| Firmicutes | 37.061^b^ | 49.563^a^ | 36.710^b^ | 1.9620 | <0.001 |
| Proteobacteria | 1.448^b^ | 0.871^c^ | 2.037^a^ | 0.1565 | <0.001 |
| Tenericutes | 1.273^b^ | 1.126^b^ | 1.805^a^ | 0.2817 | 0.011 |
| Actinobacteria | 0.882^b^ | 1.382^a^ | 0.889^b^ | 0.2941 | 0.058 |
| Verrucomicrobia | 1.161^a^ | 0.652^b^ | 1.018^a^ | 0.2238 | 0.020 |
| Saccharibacteria | 0.692^ab^ | 0.894^a^ | 0.487^b^ | 0.1760 | 0.021 |
| SR1_Absconditabacteria | 0.676 | 0.532 | 0.603 | 0.0919 | 0.784 |
| Spirochaetae | 0.529^ab^ | 0.421^b^ | 0.610^a^ | 0.0820 | 0.021 |
| Synergistetes | 0.757^a^ | 0.354^b^ | 0.350^b^ | 0.1800 | 0.009 |

^1^ HAL represents the high-altitude region (Zhongba County, Xigatse City, 4 800 m altitude), MAL represents the medium-altitude region (Nagqu City, 4 500 m altitude), LAL represents the low-altitude region (Dangxiong County, Lhasa City, 3 800 m altitude).

Different superscript letters in the same row denote significant differences (*P* < 0.05).

**Supplementary Table 2.** Composition of rumen microbiota at the family level.

| Family | Groups^1^ | | | SEM | *P*-value |
| --- | --- | --- | --- | --- | --- |
|  | HAL | MAL | LAL |  |  |
| Prevotellaceae | 0.234 | 0.235 | 0.271 | 0.011 | 0.228 |
| Ruminococcaceae | 0.152 | 0.144 | 0.146 | 0.003 | 0.496 |
| Rikenellaceae | 0.157^a^ | 0.082^c^ | 0.111^b^ | 0.007 | <0.001 |
| Lachnospiraceae | 0.084^b^ | 0.109^a^ | 0.081^b^ | 0.004 | 0.011 |
| Christensenellaceae | 0.070^b^ | 0.102^a^ | 0.081^b^ | 0.004 | 0.004 |
| Bacteroidales_BS11_gut_group | 0.081^a^ | 0.068^b^ | 0.068^b^ | 0.002 | 0.010 |
| Veillonellaceae | 0.012^b^ | 0.099^a^ | 0.011^b^ | 0.011 | 0.004 |
| unidentified | 0.042^a^ | 0.032^b^ | 0.045^a^ | 0.002 | 0.015 |
| Bacteroidales_S24-7_group | 0.032^b^ | 0.025^b^ | 0.046^a^ | 0.003 | 0.009 |
| Acidaminococcaceae | 0.022 | 0.017 | 0.020 | 0.001 | 0.367 |
| Bacteroidales_RF16_group | 0.013^b^ | 0.013^b^ | 0.020^a^ | 0.001 | 0.017 |

^1^ HAL represents the high-altitude region (Zhongba County, Xigatse City, 4 800 m altitude), MAL represents the medium-altitude region (Nagqu City, 4 500 m altitude), LAL represents the low-altitude region (Dangxiong County, Lhasa City, 3 800 m altitude).

Different superscript letters in the same row denote significant differences (*P* < 0.05).

**Supplementary Table 3.** Composition of rumen microbiota at the genus level.

| Genus | Groups^1^ | | | SEM | *P* |
| --- | --- | --- | --- | --- | --- |
|  | HAL | MAL | LAL |  |  |
| *Christensenellaceae_R-7_group* | 6.786^b^ | 9.573^a^ | 7.830^b^ | 0.890 | 0.002 |
| *Prevotellaceae_UCG-003* | 3.300^a^ | 1.903^b^ | 3.671^a^ | 0.615 | 0.002 |
| *unidentified* | 25.399^a^ | 19.802^b^ | 26.116^a^ | 1.563 | <0.001 |
| *Ruminococcaceae_NK4A214_group* | 2.695^b^ | 3.653^a^ | 2.906^b^ | 0.410 | 0.014 |
| *Rikenellaceae_RC9_gut_group* | 15.190^a^ | 7.884^c^ | 10.711^b^ | 1.214 | <0.001 |
| *Eubacterium_coprostanoligenes_group* | 1.888^a^ | 1.030^c^ | 1.413^b^ | 0.163 | <0.001 |
| *Saccharofermentans* | 1.457^a^ | 0.951^b^ | 1.380^a^ | 0.163 | 0.001 |
| *Ruminococcaceae_UCG-010* | 1.296^a^ | 0.946^b^ | 0.923^b^ | 0.132 | 0.002 |
| *Prevotellaceae_NK3B31_group* | 1.220^a^ | 0.379^b^ | 1.480^a^ | 0.265 | <0.001 |
| *Ruminococcaceae_UCG-005* | 0.982^b^ | 1.285^a^ | 0.619^c^ | 0.178 | <0.001 |
| *Papillibacter* | 0.907^b^ | 0.632^b^ | 1.697^a^ | 0.175 | <0.001 |
| *Quinella* | 0.745^b^ | 8.865^a^ | 0.594^b^ | 2.451 | <0.001 |
| *Butyrivibrio_2* | 0.574^c^ | 1.056^a^ | 0.814^b^ | 0.147 | <0.001 |
| *Olsenella* | 0.364^b^ | 0.972^a^ | 0.531^b^ | 0.240 | 0.009 |
| *Lachnospiraceae_NK3A20_group* | 0.310^b^ | 2.747^a^ | 0.295^b^ | 0.227 | <0.001 |
| *Acetitomaculum* | 0.189^b^ | 1.321^a^ | 0.209^b^ | 0.160 | <0.001 |

^1^ HAL represents the high-altitude region (Zhongba County, Xigatse City, 4 800 m altitude), MAL represents the medium-altitude region (Nagqu City, 4 500 m altitude), LAL represents the low-altitude region (Dangxiong County, Lhasa City, 3 800 m altitude).

Different superscript letters in the same row denote significant differences (*P* < 0.05).


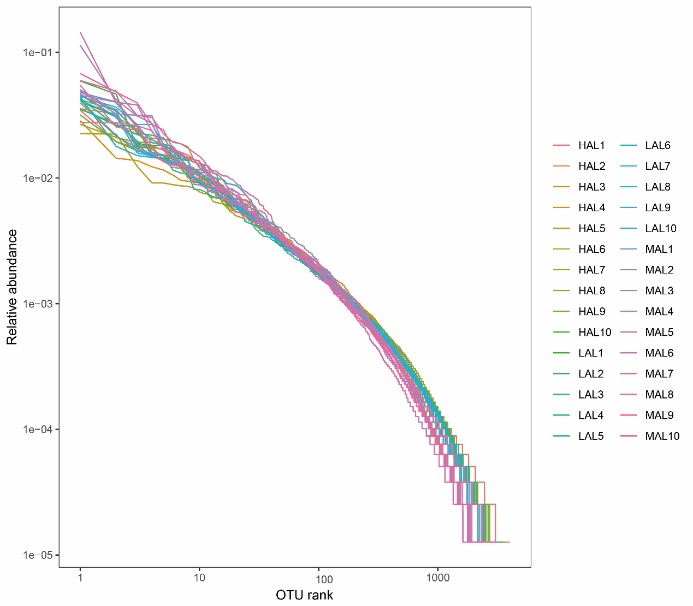


**Supplementary Fig.1**Rank abundance curves based on the OUT level.
